# Dynamic activity of human brain task-specific networks

**DOI:** 10.1038/s41598-020-64897-2

**Published:** 2020-05-12

**Authors:** Jie Huang

**Affiliations:** Department of Radiology, Michigan State University, East Lansing, MI USA

**Keywords:** Neural circuits, Dynamic networks

## Abstract

A simple motor behaviour or a more complex behaviour is the result of the neural activity of those neural networks responsible for the behaviour. To understand how the network activity is transformed into human behaviours, it is necessary to identify task-specific networks and analyse the dynamic network activity that changes with time. Here we report a novel task-fMRI technique to identify task-specific networks and analyse their dynamic activity. Nine subjects participated in a task-fMRI study in which the subjects were cued to perform three different tasks of word-reading, pattern-viewing and finger-tapping. A functional area of unitary pooled activity (FAUPA) is defined as an area in which the temporal variation of the activity is the same across the entire area, and a task-associated FAUPA plays the role of a functional unit for the task. A novel method is presented to (1) identify FAUPAs that are associated with each task as well as their functional groupings; (2) identify the three task-specific networks; and (3) analyse the network activity from trial to trial. Task-associated FAUPAs were identified for each task and each subject. A task-specific large-scale neural network across the whole brain consisted of all FAUPAs that were activated each time the task was performed, and all three task-specific networks were identified for each individual subject. The temporal changes of activation and functional connectivity of the FAUPAs within each network from trial to trial characterized the dynamic activity of the network. The results demonstrated a one-to-one relation between the network activity and the task performance from trial to trial, offering a means of testing the causal relationship between network activity and human task performance by systematically manipulating task performance and measuring corresponding network activity change.

## Introduction

To understand the relationship between the neural network activity and human behaviours, it is necessary first to identify functional networks specific for tasks and analyse the dynamic network activity when performing these tasks. A task-specific network should be composed of a set of functional areas that are activated when the task is performed, and each functional area may play a role of functional unit responsible for the specific function. It is essential to identify task-associated functional areas and task-specific networks for investigating the causal relationship between the neural network activity and human behaviours.

The blood oxygenation level dependent (BOLD) functional magnetic resonance imaging (fMRI) technique provides a non-invasive neuroimaging tool to investigate brain activity in terms of large-scale neuron population dynamics across the whole brain^1–3^. The BOLD-fMRI technique has the potential to identify task-specific networks at a large-scale, but the conventional approaches of analysing task-fMRI data lack the capability of identifying functionally unitary areas responsible for the performance of tasks. The general linear model (GLM) is currently the most popular statistical approach to identifying brain areas activated by a task^4,5^. This model sets up an expected ideal response and fits it with the BOLD signal time course on a voxel-by-voxel basis so as to generate a map of t or z statistics. Then, a threshold with a chosen significance level is used to identify the activated voxels. The areas identified depend on the threshold chosen, and different thresholds yield different areas. Accordingly, such an identified activated area most likely does not play the role of functional unit responsible for the specific function. Consider a simple finger-tapping task we should expect to identify: (1) an activated area in the finger representation area in the primary motor cortex (M1) and (2) an activated area in the finger representation area in the primary somatosensory cortex (S1). The GLM would correctly identify a large activated area in the sensorimotor cortex, but it could not differentiate these two activated areas in M1 and S1 because the temporal variation of the BOLD response is similar in these two areas. In other words, an activated area identified by the GLM may consist of two or more functionally unitary areas specific for different but associated functions. As the temporal variation of the BOLD response is similar in these two areas, other conventional approaches such as the independent component analysis (ICA) and principal component analysis (PCA)^6,7^ also could not differentiate them in M1 and S1, and therefore suffer the same problem. To map task-specific networks requires the identification of those functionally unitary areas that compose the networks. Accordingly, novel approaches need to be developed to map human brain task-specific networks. In addition, the GLM assumes that the expected ideal response accurately models the task-evoked BOLD response. The GLM is a good model for analysing task-fMRI data as demonstrated by numerous studies. However, for those individuals with difficulty to perform a task properly, the expected ideal response generated in the GLM could deviate from the task-evoked BOLD response substantially, rendering the GLM analysis in error.

The discovery of functional areas of unitary pooled activity (FAUPAs) with fMRI was recently reported^8^, and a FAUPA-based novel method was developed to compare the brain activity between the resting state and task state^9^. A FAUPA was defined as an area in which the temporal variation of the activity was the same across the entire area, and new techniques were developed to identify FAUPAs that involved the iterative aggregation of voxels dependent upon their intercorrelation^10^. FAUPAs are determined objectively and automatically, based on the assumption that the temporal variation of the activity is the same across the entire area within a FAUPA. Unlike the GLM, a priori knowledge of the activity-induced ideal response signal time course is not required for FAUPA determination. Identifying the cortical locations of FAUPAs or comparing their signal time courses with a presumed task-induced ideal response may enable us to identify those that are associated with a specific task. Using the signal time course of a task-associated FAUPA may identify the functional network specific for that task. In this study nine subjects undertook a task-fMRI scan in which they performed three tasks of word-reading (WR), pattern-viewing (PV) and finger-tapping (FT). We present a FAUPA-based novel method to identify task-specific networks and analyse the dynamic network activity of these networks. The BOLD signal time courses of these task-associated FAUPAs may characterize the dynamic activity of the task-specific network that changes from trial to trial, resulting in a one-to-one relationship between a network activity and the task performance from trial to trial.

## Results

### Finger-tapping associated FAUPAs in the primary motor and somatosensory cortices

**Figure 1:**
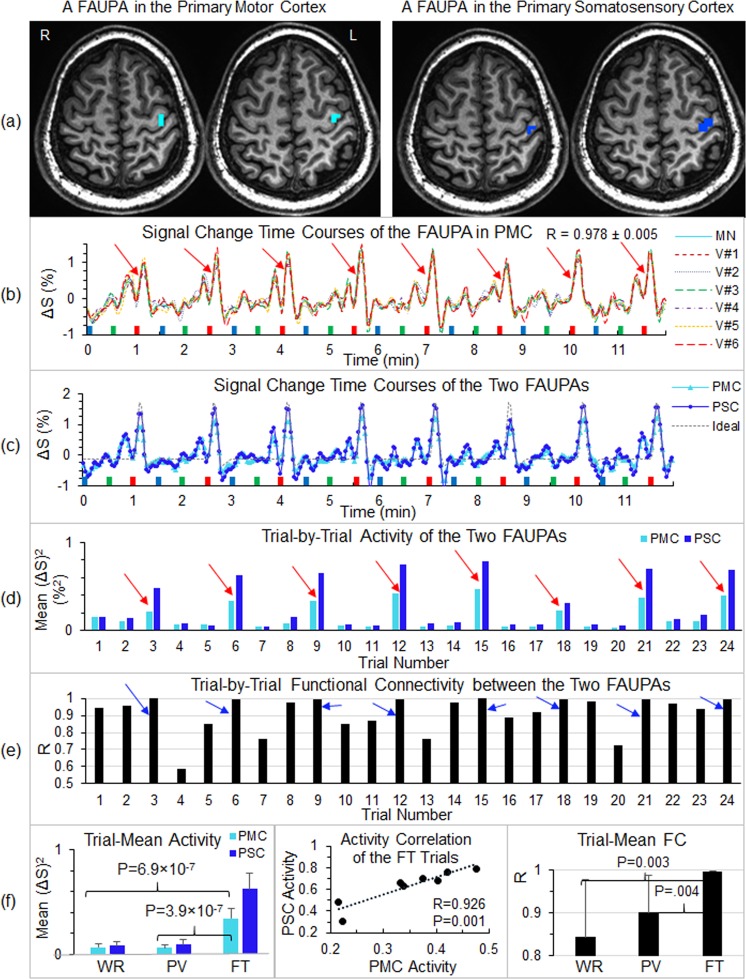
Illustration of two finger-tapping (FT) associated FAUPAs in the left sensorimotor cortex for a representative subject. (**a**) The cyan cluster represents one FAUPA in the primary motor cortex (PMC) and the blue cluster represents the other one in the primary somatosensory cortex (PSC). R: right, and L: left; (**b**) The FAUPA in PMC comprised the six cyan voxels, and their signal change time courses were very similar to each other. The task paradigm consisted of 24 tasks shown by the 24 bars: blue bars representing word-reading (WR), green bars pattern-viewing (PV) and red FT. Each task lasted 6 s followed by 24 s rest, forming one task trial. A task-induced large signal change that was time-locked with each FT task (red bar) is conspicuous for all eight FT trials (eight red arrows). V: voxel, MN: mean; (**c**) Comparison of the signal change time courses of these two FAUPAs as well as the ideal response; (**d**) Comparison of the trial-by-trial activity of these two FAUPAs for the twenty-four trials. The neural activity was measured as the BOLD signal change squared at each time point^20^, and each bar represents the mean activity averaged over each trial period. Eight red arrows point out the large neural activity evoked by the eight FT trials; (**e**) Comparison of the trial-by-trial functional connectivity (FC) (measured with Pearson correlation coefficient R) between these two FAUPAs. Eight blue arrows point out an increased FC during each of the eight FT trials; and (**f**) Comparison of the trial-mean activity (mean activity over the eight trials) for the three tasks (left), correlation of the FT-induced activation between these two FAUPAs for the 8 FT trials (middle), and the trial-mean FC between these two FAUPAs for the three tasks (right). The error bars represent the standard deviations.

For the FT task, the subjects were cued to tap the five fingers of their right-hand on the keypad as quickly as possible in a random order. Accordingly, the execution of the FT task should induce an associated FAUPA in the left primary motor cortex (M1) due to the execution of the finger movements, and an associated FAUPA in the left primary somatosensory cortex (S1) that reflects the somatic sensation when tapping the fingers, respectively. Figure 1a illustrates two FT-associated FAUPAs for a representative subject; one FAUPA was located precisely in the left M1 and the other (completely separate area) was located precisely in the left S1, respectively. Their signal change time courses showed an almost identical temporal behaviour (R = 0.973, P = 3.1 × 10^−61^ for N = 288) except as regards their activation magnitudes, and a FT-induced large signal change was conspicuous for each of the eight FT trials (Fig. 1c). These signal change time courses also matched very well with the ideal response time course of the FT task alone, showing their association with the FT task. In this study, we used the mean of the squared relative signal changes over each trial period to quantify the FAUPA activity as a function of task trial, and a FT-induced substantial increase in the FAUPA activity was demonstrated for each of the eight FT trials as illustrated in Fig. 1d. For the FAUPA in M1, the mean activity averaged over the eight trials was 0.347 ± 0.092 (%^2^) for FT, 0.066 ± 0.039 (%^2^) for WR, and 0.063 ± 0.027 (%^2^) for PV, respectively. This activity for FT was significantly larger than that for either WR (one-tail t-test, p = 6.9 × 10^−7^) or PV (p = 3.9 × 10^−7^) (Fig. 1f, left), but there was no statistical difference between WR and PV (two-tail t-test, p = 0.88). Similar results were obtained for the FAUPA in S1. These results demonstrate significantly increased neural activity in these two FAUPAs when performing the FT task compared to that when performing the other two tasks. Comparing these two FAUPAs, their FT-induced activity further showed a significant correlation with each other for the eight FT trials (R = 0.9259, p < 0.001) (Fig. 1f, middle). Figure 1e plots the corresponding functional connectivity of these two FAUPAs from trial to trial. This functional connectivity was statistically significant for each of the 24-trials (the minimum R = 0.5864, p = 0.045), showing that the functional connectivity between these two FAUPAs was statistically significant during each trial period regardless of the task. In comparison to the WR and PV tasks, however, the functional connectivity was increased substantially during each FT task as shown in Fig. 1e. The trial-mean R was 0.997 ± 0.002 for FT, 0.844 ± 0.134 for WR, and 0.901 ± 0.086 for PV, respectively. The R for FT was significantly larger than that of either WR (one-tail t-test, p = 0.003) or PV (p = 0.004), but there was no statistical difference between WR and PV (two-tail t-test, p = 0.33) (Fig. 1f, right). It demonstrates that the functional connectivity was increased significantly during the FT task compared to the other two tasks. The trial-mean R of 0.997 demonstrates an almost perfect synchronization of the activity in these two FAUPAs during the performance of the FT task. The temporal changes of the activity of these two FAUPAs and of the functional connectivity between them as a function of task trial show the dynamic activity of these two FT-associated FAUPAs from trial to trial (Fig. 1d,e).

### Comparison of the finger-tapping-evoked activation maps

**Figure 2:**
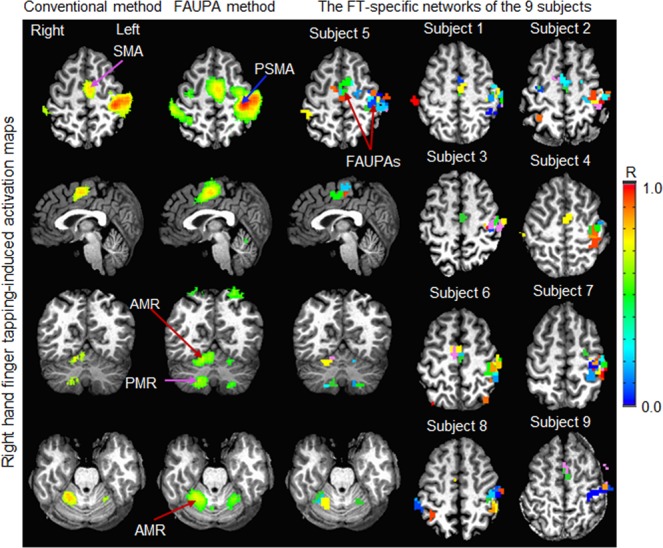
Comparison of the group mean finger-tapping (FT) evoked activation maps. Left column: the FT-activated areas generated with the GLM; 2^nd^ column: the FT-activated areas generated with the FAUPA method; 3^rd^ column: the FT-specific network of the representative subject; and the last two columns: the FT-specific networks of the other 8 subjects in the same slice in the standard template space. (Note that each subject’s anatomical structure is presented to illustrate the structure variation from subject to subject.) In the last three columns, each colored cluster with all connected voxels of the same color represented a FAUPA, and a total of 16 colors was used to differentiate these FAUPAs. The large FT-activated area in the left primary sensorimotor area (PSMA), generated with the GLM, was separated into multiple FT-associated FAUPAs. The same was true for the supplementary motor area (SMA), anterior motor region (AMR) of the cerebellum and posterior motor region (PMR) of the cerebellum. A similar FT-specific network was observed for every subject, and the same slice in the standard template space was selected to illustrate the FT-associated FAUPAs in both PSMA and SMA (the last two columns). As the anatomical structure varied substantially across the subjects, the FT-associated FAUPAs in the SMA did not appear in subjects 7 and 8 because they were located on the adjacent slices. Tapping right-hand fingers evoked not only the familiar contralateral cerebrocerebellar circuit (i.e., the left cerebral motor areas and the right cerebellar motor regions), but also the ipsilateral cerebrocerebellar circuit (i.e., the right cerebral motor areas and the left cerebellar motor regions), at both individual and group levels (2^nd^ and 3^rd^ columns), though the latter was much weaker than the former. Note that, in contrast, the GLM only identified the contralateral cerebrocerebellar circuit.

The right-hand FT-evoked activation map, generated with the GLM, showed large activated clusters in the left sensorimotor cortex, the left supplementary motor area, and the right anterior and posterior motor regions of the cerebellum, i.e., the familiar contralateral cerebrocerebellar circuit with respect to the cerebrum (i.e., the left M1 controls the movement of the right-hand fingers) (Fig. 2, left column). Using the signal time course of the selected FT-associated FAUPA in the left M1 (Fig. 1a,b) generated a similar FT-evoked activation map (Fig. 2, 2^nd^ column). This result is expected because the signal time course of the selected FAUPA was very similar to the ideal response time course of the FT task alone (Fig. 1c). Even when performing the simple FT task, the task-induced BOLD response varied substantially from trial to trial (Fig. 1b,c) and from subject to subject (data not shown). One advantage of using this FAUPA method was that using the signal time course of the selected FAUPA took account of this variation for each individual, yielding a more accurate FT-evoked activation map as evidenced in the 2^nd^ column of Fig. 2. Tapping the fingers of the right-hand evoked not only the expected contralateral cerebrocerebellar circuit, but also the unexpected ipsilateral cerebrocerebellar circuit, which was revealed with the FAUPA method but not the GLM.

### Individual finger-tapping specific network

**Figure 3:**
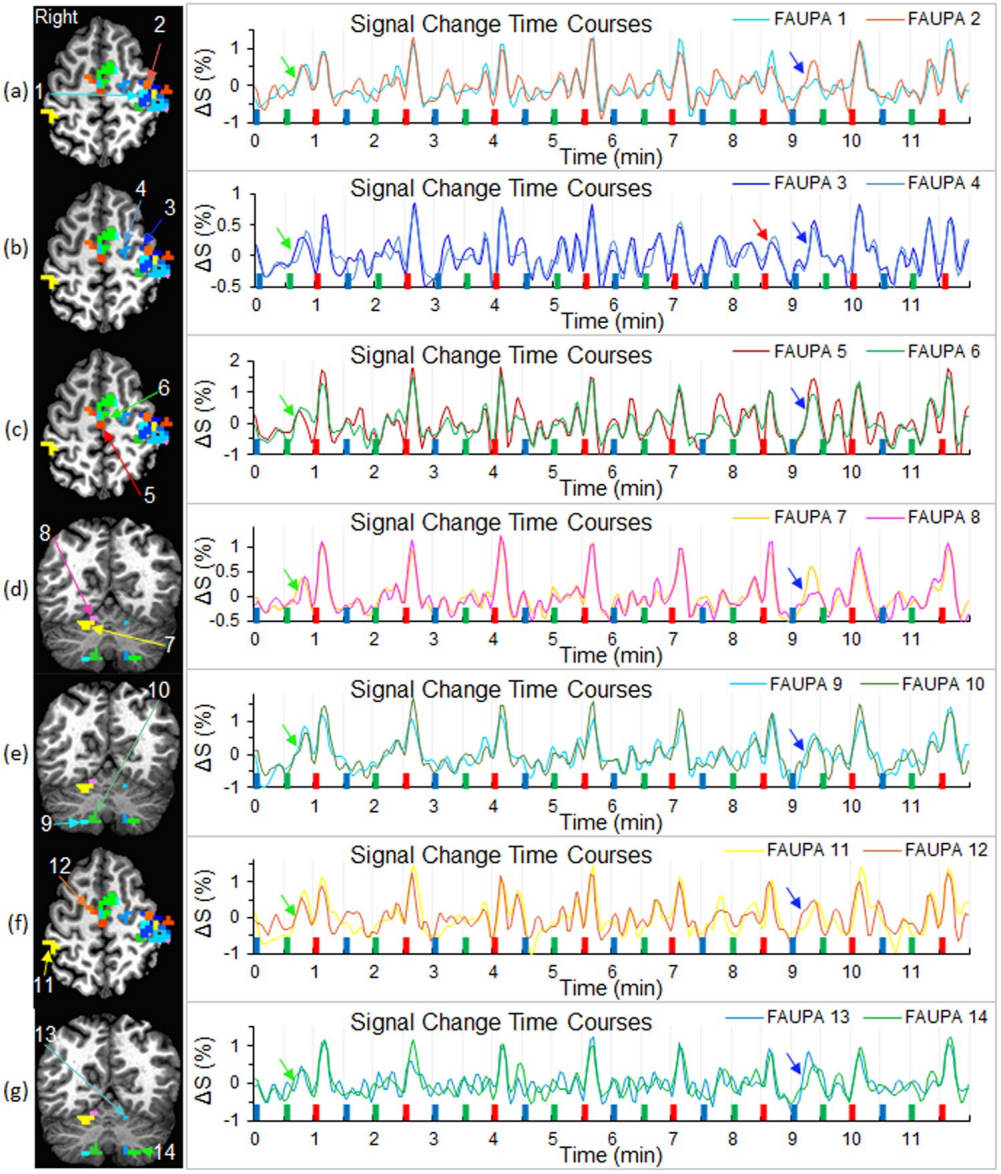
Comparison of the FT-induced activation across the FT-specific network for a representative subject. A total of 14 FAUPAs were selected to illustrate the right-hand FT-evoked activation across both contralateral and ipsilateral cerebrocerebellar circuits. The FT-induced activation of the contralateral cerebrocerebellar circuit was depicted by FAUPAs from 1 to 10: (**a**) left primary motor cortex (FAUPAs 1 and 2); (**b**) left precentral gyrus (FAUPAs 3 and 4); (**c**) left supplementary motor area (FAUPAs 5 and 6); (**d**) right anterior motor region of the cerebellum (FAUPAs 7 and 8); and (**e**) right posterior motor region of the cerebellum (FAUPAs 9 and 10). The FT-induced activation of the ipsilateral cerebrocerebellar circuit was depicted by FAUPAs from 11 to 14: (**f**) right postcentral gyrus (FAUPA 11) and right supplementary motor area (FAUPA 12); and (**g)** left anterior motor region of the cerebellum (FAUPA 13) and left posterior motor region of the cerebellum (FAUPA 14). For each paired FAUPAs from (**a)** to (**g**), the right plot compares the similarity and difference of the signal time course between these two FAUPAs. Note that some of these paired FAUPAs (e.g., FAUPAs 1 and 2) are adjacent to each other, but they may represent two different functional units. Each FT task (eight red bars) induced a large signal change, and this FT-evoked signal change processed across both contralateral and ipsilateral cerebrocerebellar circuits.

For each subject we used the signal time course of the selected FAUPA in the primary motor cortex to identify FAUPAs associated with the FT task, and the 3^rd^ column of Fig. 2 illustrates these FT-associated FAUPAs for the representative subject. It demonstrates that each large activated clusters in the 1^st^ and 2^nd^ columns were separated into 2 or more FAUPAs. Similar results were observed for all other eight subjects (Fig. 2, last two columns). All identified FT-associated FAUPAs composed a FT-specific network for each subject. For the representative subject Fig. 3 illustrates the FT-induced signal changes from trial to trial across the FT-specific network. A right-hand FT-induced large signal change that was time-locked with each FT task is conspicuous for all eight FT trials across both contralateral and ipsilateral cerebrocerebellar circuits. The degree of co-activity within each circuit and between these two circuits was measured with correlation coefficient R, and these R values were tabulated in Table 1. The FT-induced signal change varied from trial to trial. For example, the signal change evoked by the 6^th^ FT task was substantially smaller for FAUPAs 3 and 4 (the red arrow in Fig. 3b) compared to those other FAUPAs, demonstrating a dynamic FT-induced signal change from trial to trial across the FT-specific network *that was one-to-one related with the performance of each of the eight FT trials*. A noticeable time-locked signal change in the first PV trial occurred consistently across all these fourteen FAUPAs (seven green arrows in Fig. 3), suggesting a neural event that processed across the whole network, though its nature was unknown. Another noticeable signal change in the 7^th^ WR trial also occurred consistently across all these FAUPAs (seven blue arrows in Fig. 3), suggesting another neural network event although again its nature was unknown. These consistently time-locked signal changes across the network suggest they were finger movement related activities but not noise, though we did not monitor the subject’s finger movements through the scan. They provided further evidence to show that the presented FAUPA method may be capable of detecting trial by trial activations and linking them with behaviours trial by trial at an individual level.

**Table 1:** Comparison of co-activation both within and between the right-hand finger-tapping evoked contralateral and ipsilateral cerebrocerebellar circuits (CCCs) with respect to the cerebrum (i.e., the right hand corresponds to the left cerebrum).

	FAUPAs	Pearson Correlation Coefficient R						
		Contralateral CCC					Ipsilateral CCC	
		1&2 L M1	3&4 L PCG	5&6 L SMA	7&8 R AMR	9&10 R PMR	11&12 R SMA	13&14 L A&PMRs
Contralateral CCC	1&2 L M1	1	0.893	0.887	0.874	0.897	0.893	0.863
	3&4 L PCG	0.893	1	0.872	**0.726**	0.795	0.861	0.762
	5&6 L SMA	0.887	0.872	1	0.806	0.812	0.899	0.833
	7&8 R AMR	0.874	**0.726**	0.806	1	0.919	0.818	0.886
	9&10 R PMR	0.897	0.795	0.812	0.919	1	0.875	0.872
Ipsilateral CCC	11&12 R SMA	0.893	0.861	0.899	0.818	0.875	1	0.860
	13&14 L A&PMRs	0.863	0.762	0.833	0.886	0.872	0.860	1

For each paired FAUPAs in Fig. 3, the mean signal time course was computed and then the correlation coefficient was computed between those seven paired FAUPAs. The minimum R = 0.726, and the corresponding maximum P = 7.0 × 10^−35^ for N = 288. L: left; R: right; M1: primary motor area; PCG: precentral gyrus; SMA: supplementary motor area; AMR: anterior motor region; PMR: posterior motor region; and A&PMRs: anterior and posterior motor regions.

### Task-specific networks

**Figure 4:**
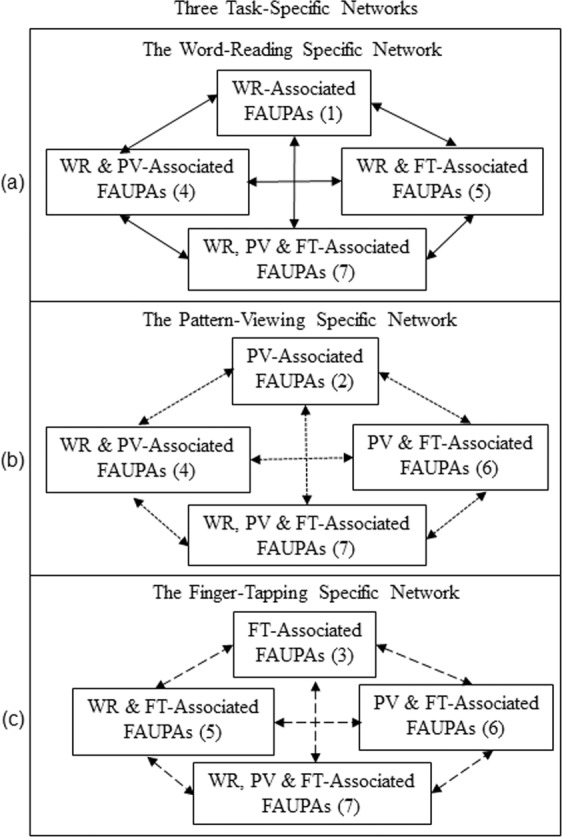
Illustration of the three task-specific networks. The word-reading (WR) specific network is described by the solid-line linked FAUPAs in (**a**), the pattern-viewing (PV) specific network by the dotted-lines in (**b**), and the finger-tapping (FT) specific network by the dashed-lines in (**c**), respectively. There are four sets of task-associated FAUPAs for each network as illustrated. All FAUPAs associated with one task alone constitute the functional units that activate only when the task is performed. All FAUPAs associated with two tasks constitute the functional units that activate when performing either task. Each FAUPA associated with all three tasks constitutes a functional unit that activates for all three tasks.

**Figure 5:**
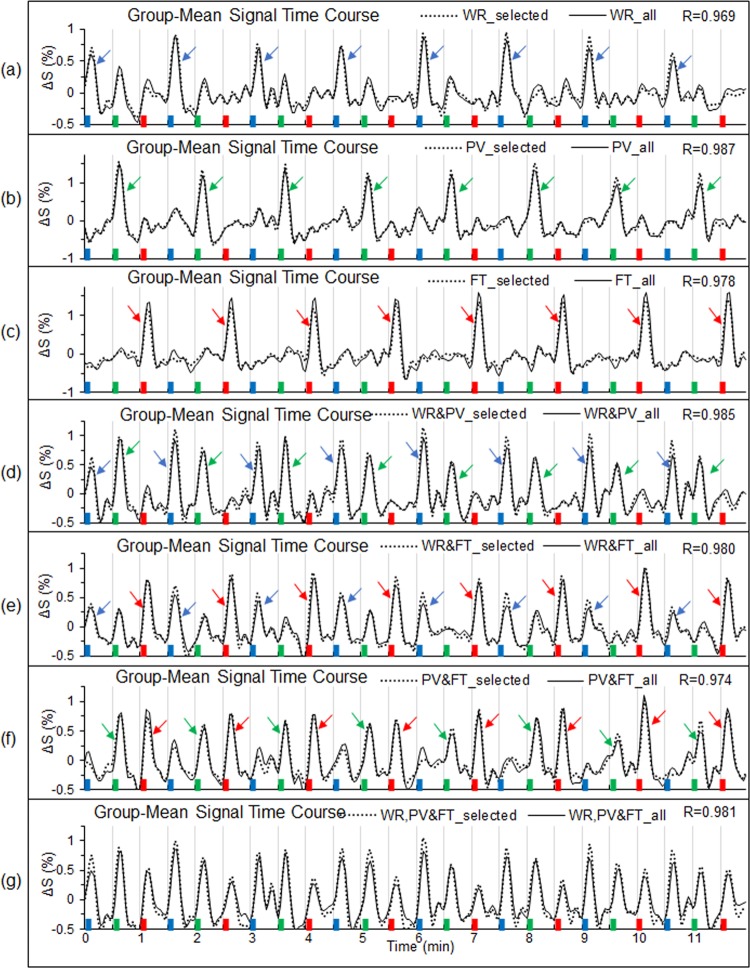
Comparison of the group-mean signal time courses between the selected FAUPAs and all the task-associated FAUPAs for each of the seven categories of the task-associated FAUPAs (Fig. 4). In each panel, the dotted-line represents the group-mean signal time course of the selected FAUPA that was associated with the corresponding task(s), and the solid line represents the group-mean signal time course of all task-associated FAUPAs, respectively. For example, the dotted line in (**c**) was the mean signal time course of the 9 selected FAUPAs (one from each subject), and these FAUPAs were located in the similar primary motor cortex for all subjects (Fig. 1). The solid line in (**c**) was the mean signal time course of the 416 FAUPAs associated with the finger-tapping task alone (Table 2), and these FAUPAs are located across the whole brain (Figs. 2 and 3). The blue, green and red bars represent the onset and duration of the three tasks of WR, PV and FT, respectively. For each category, a task-evoked time-locked signal change is conspicuous for each task trial, showing the association of these FAUPAs with the task. For a better visual comparison of the similarity and difference of these signal time courses, their corresponding standard deviations were not added.

A task-associated FAUPA is defined as a FAUPA that is activated when the task is performed. There were three different tasks in this study, and therefore there were a total of seven functional groupings, i.e., seven categories of the task-associated FAUPAs, forming three task-specific networks as depicted in Fig. 4. For each subject, a representative task-associated FAUPA was identified for each of the seven categories, and its corresponding group-mean signal time course of the selected FAUPA averaged over the nine subject was plotted in Fig. 5. For each category, the group-mean signal time course demonstrated that the FAUPA was activated each time the task was performed, showing the association of the FAUPA with the task. For each subject and each category, the signal time course of the selected FAUPA was used to compute the R-value for all FAUPAs and task-associated FAUPAs were identified with the threshold R > 0.8 (N = 288, P = 5.5 × 10^−42^). Table 2 tabulates these identified task-associated FAUPAs for each category and each subject. The total number of the identified task-associated FAUPAs varied substantially from subject to subject for each category. To examine the overall behaviour of the task-evoked signal changes across the three task-specific networks, for each subject we first computed the mean signal time course averaged over all those task-associated FAUPAs for each category, and then computed their group-mean signal time course averaged over the nine subjects for that category. This group-mean signal time course was almost identical to that of the selected FAUPAs for each category, showing a remarkably similar task-evoked activity processed across the whole network for each of the three task-specific networks when that task was performed (Fig. 5). For each of the three task-specific networks, every FAUPA constituting that network was activated each time when that task was performed, i.e., the network was activated from trial to trial, but the other two networks remained relatively “silent”, yielding a one-to-one relation between the network activity and the task performance.

**Table 2:** Number of task-associated FAUPAs identified for each category in Fig. 5 and each subject. WR: word-reading; PV: pattern-viewing; FT: finger-tapping; MN: mean; and SD: standard deviation.

Subject	Number of FAUPAs							
	WR	PV	FT	WR&PV	WR&FT	PV&FT	WR,PV&FT	Total
1	15	29	56	5	25	37	47	214
2	8	13	71	5	2	3	7	109
3	13	43	23	5	16	10	5	115
4	2	25	13	18	4	10	3	75
5	3	23	74	11	2	4	7	124
6	5	34	43	37	17	27	7	170
7	123	2	42	76	30	48	44	365
8	2	6	43	8	3	7	5	74
9	8	27	51	6	27	49	11	179
sum	179	202	416	171	126	195	136	1425
MN	19.9	22.4	46.2	19	14	21.7	15.1	158.3
SD	38.9	13.2	20.0	23.8	11.6	18.9	17.4	90.8

### Dynamic task-evoked activity across the task-specific networks

**Figure 6:**
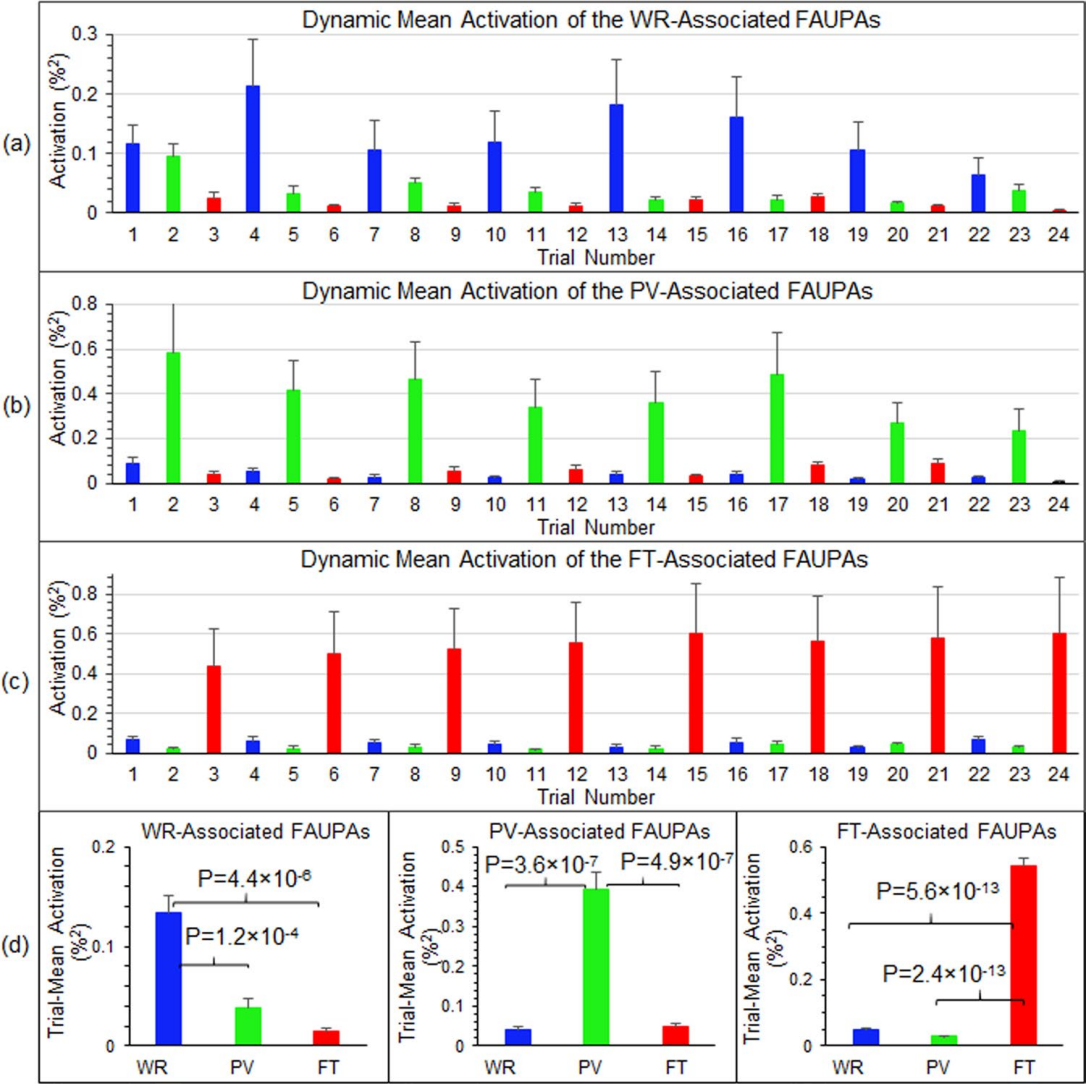
Dynamic mean activation of task-associated FAUPAs. For each of the first three categories in Fig. 5, for each FAUPA within that category we first computed a mean activation for each trial period, and then computed the mean activation over all these FAUPAs to quantify the dynamic mean activation from trial to trial for that category. We further computed the group-mean of this mean FAUPA activation from trial to trial averaged over the nine subjects for the WR-associated FAUPAs (**a**), the PV-associated FAUPAs (**b**), and the FT-associated FAUPAs (**c**), respectively. For each category from a to c, the eight same colored bars indicate their corresponding task-induced large activation. (**d**) Comparison of the trial-mean activation averaged over the eight trials for each category of the three task-associated FAUPAs. The error bars represent the standard errors of the means.

**Figure 7:**
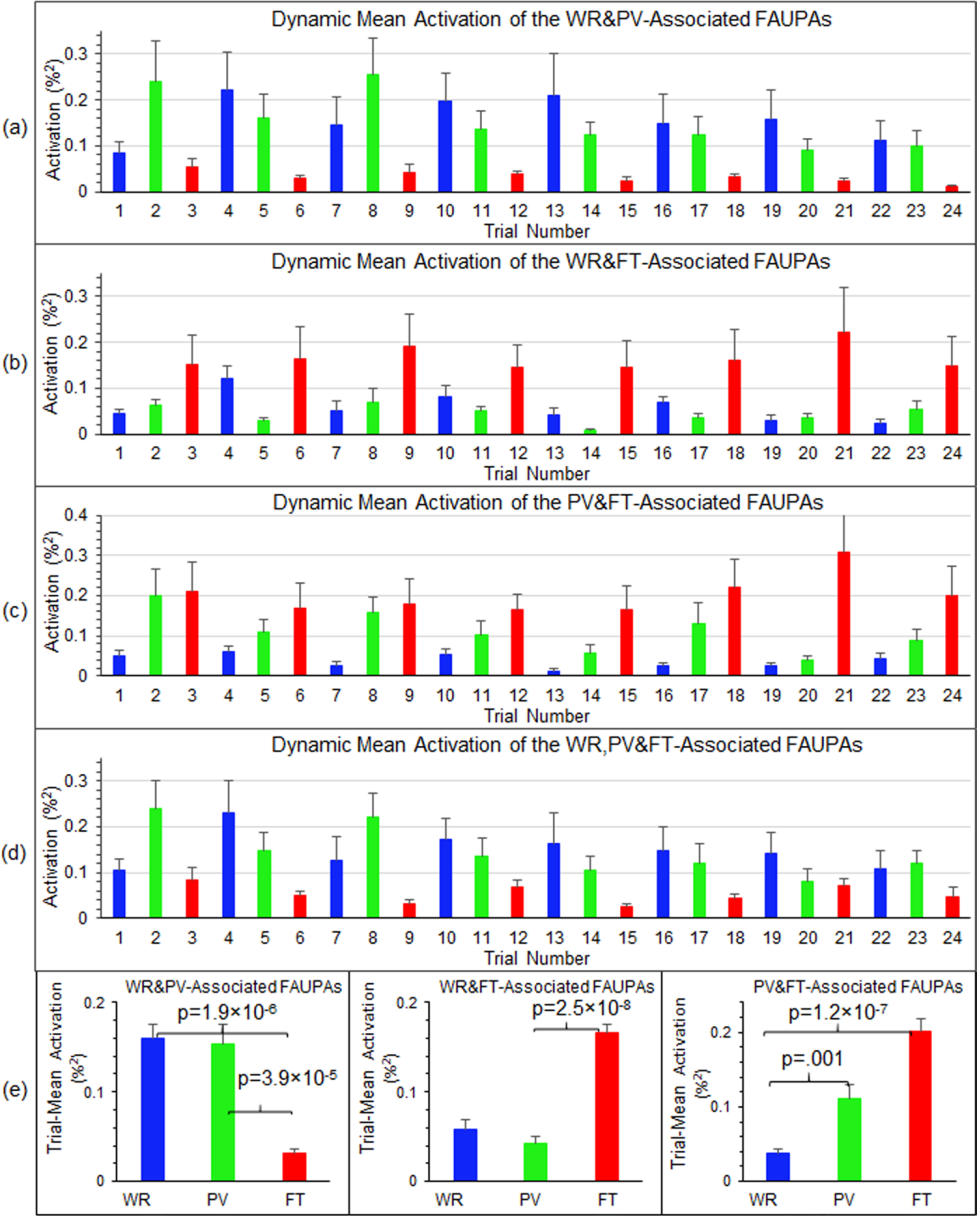
Dynamic mean activation of task-associated FAUPAs. For each of the last four categories in Fig. 5, for each FAUPA within that category we first computed a mean activation for each trial period and each FAUPA within that category, and then computed the mean activation over all these FAUPAs to quantify the dynamic mean activation from trial to trial for that category. We further computed the group-mean of this mean FAUPA activation from trial to trial averaged over the nine subjects for the WR- and PV-associated FAUPAs (**a**), the WR- and FT-associated FAUPAs (**b**), the PV- and FT-associated FAUPAs (**c**), and WR-, PV- and FT-associated FAUPAs (**d**), respectively. (**e**): Comparison of the trial-mean activation averaged over the eight trials for each category of the three corresponding task-associated FAUPAs. The error bars represent the standard errors of the means.

To further illustrate the dynamic network activity, Fig. 6a–c show the group-mean activation from trial to trial for the WR-associated, PV-associated, and FT-associated FAUPAs (i.e., the first three categories in Fig. 5), respectively. For the FT-associated FAUPAs, the mean activation for each of the eight FT trials was substantially larger than that for each of the other sixteen not-FT trials as shown in Fig. 6c. Similar behaviours of the mean activation from trial to trial were also demonstrated for the other two WR- and PV-associated FAUPA categories as shown in Fig. 6a,b. To further compare these mean activations, we computed the trial-mean activation over the eight trials for each task type, and Fig. 6d shows the results for the three categories. For the FT-associated FAUPAs, the activation for the FT trials was significantly enhanced compared to that for either the WR trials or the PV trials (one-tail t-test, max p = 5.6 × 10^−13^). Similar results were obtained for the other two WR- and PV-associated FAUPAs as shown in Fig. 6d. For each category of (4), (5) and (6), the corresponding trial-mean activation also demonstrated an expected behaviour for that category (Fig. 7). For example, for the FAUPAs associated with both WR and PV, the activation for either the WR trials or the PV trials was significantly enhanced compared to that for the FT trials (one-tail t-test, max p = 3.9 × 10^−5^) (Fig. 7e, left).

**Figure 8:**
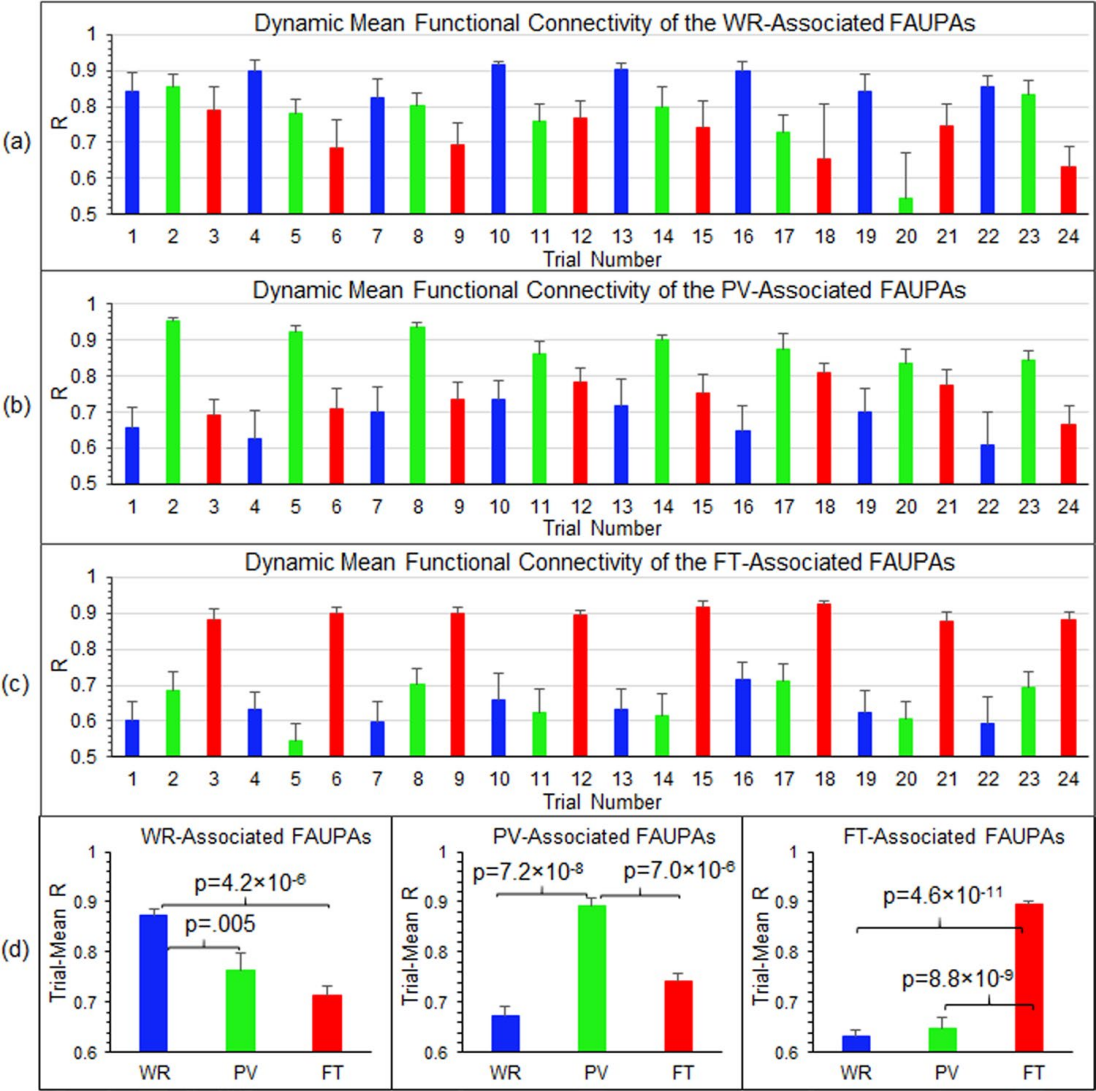
Dynamic mean functional connectivity of task-associated FAUPAs. For each of the first three categories in Fig. 5, we first computed the correlation of the signal time courses of any paired FAUPAs within that category over each trial period to quantify the functional connectivity change from trial to trial. We then computed the mean of these functional connectivity changes over all paired FAUPAs to quantify the mean functional connectivity from trial to trial for that category. We further computed the group-mean functional connectivity from trial to trial averaged over the nine subjects for the WR-associated FAUPAs (**a**), PV-associated FAUPAs (**b**), and FT-associated FAUPAs (**c**), respectively. For each category from a to c, the eight same colored bars indicate their corresponding task-increased functional connectivity. (**d**) Comparison of the trial-mean functional connectivity averaged over the eight trials for each category of the three task-associated FAUPAs. The error bars represent the standard errors of the means.

**Figure 9:**
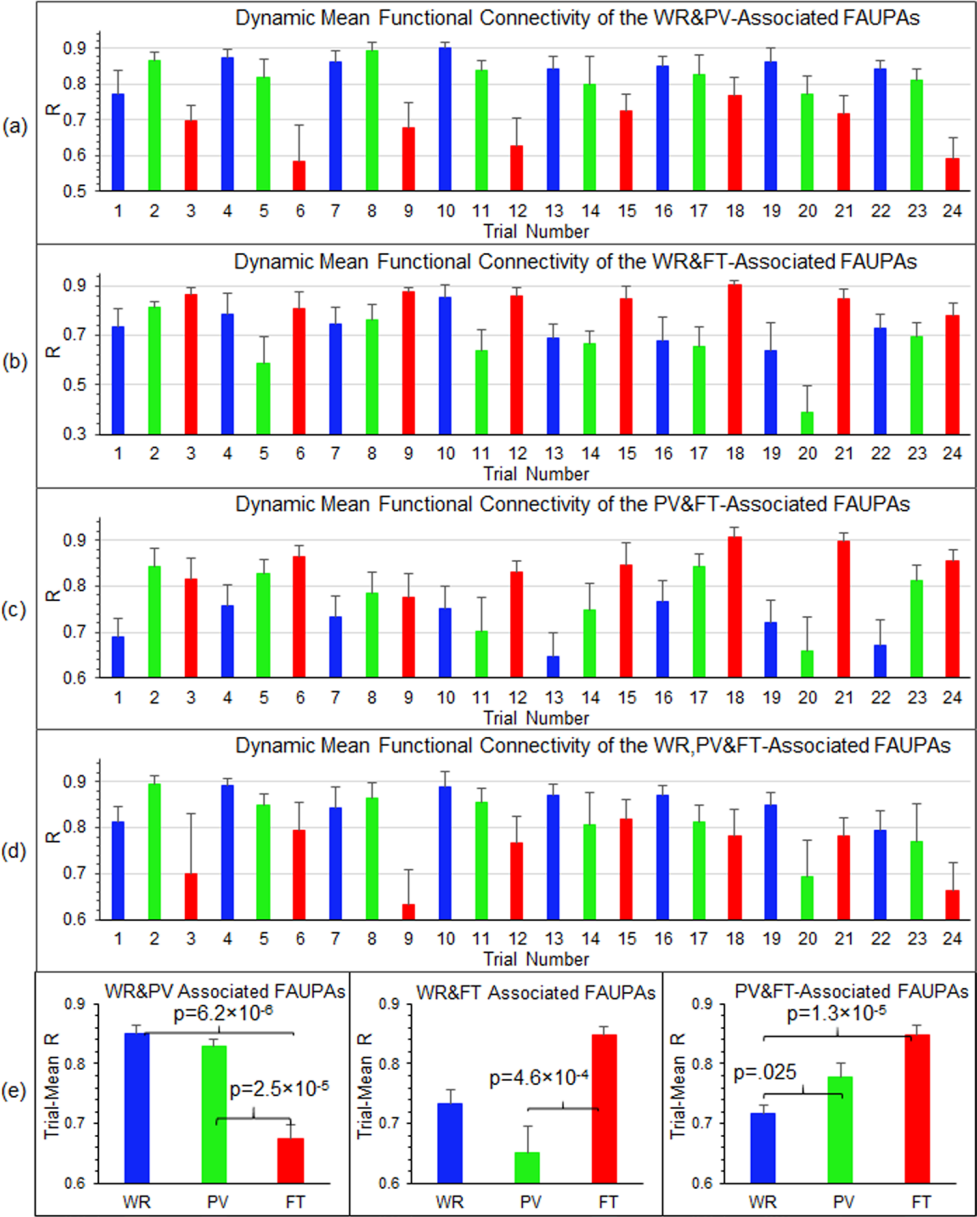
Dynamic mean functional connectivity of task-associated FAUPAs. For each of the last four categories in Fig. 5, we first computed the correlation of the signal time courses of any paired FAUPAs within that category over each trial period to quantify the functional connectivity change from trial to trial. We then computed the mean of these functional connectivity changes over all paired FAUPAs to quantify the mean functional connectivity from trial to trial for that category. We further computed the group-mean functional connectivity from trial to trial averaged over the nine subjects for the WR- and PV-associated FAUPAs (**a**), WR- and FT-associated FAUPAs (**b**), PV- and FT-associated FAUPAs (**c**), and WR-, PV- and FT-associated FAUPAs (**d**), respectively. (**d**) Comparison of the trial-mean functional connectivity averaged over the eight trials for each category of the three corresponding task-associated FAUPAs. The error bars represent the standard errors of the means.

To illustrate the dynamic co-activity across each network, for each category we also computed the correlation of the signal time courses of any paired FAUPAs within that category over each trial period to quantify the functional connectivity change from trial to trial. We then computed the mean of these functional connectivity changes over all paired FAUPAs to quantify the mean functional connectivity from trial to trial for that category. Figure 8a–c shows the group-mean functional connectivity from trial to trial averaged over the nine subjects for the WR-associated, PV-associated, and FT-associated FAUPAs, respectively. For the FT-associated FAUPAs, the mean functional connectivity for each of the eight FT trials was substantially larger than that for each of the other sixteen not-FT trials as shown in Fig. 8c. Similar behaviours of the mean functional connectivity from trial to trial were also demonstrated for the other two WR- and PV-associated FAUPA categories as shown in Fig. 8a,b. To further compare these mean functional connectivity changes, we computed the trial-mean functional connectivity over the eight trials for each task type, and Fig. 8d shows the results for the three FAUPA categories. For the FT-associated FAUPAs, the functional connectivity for the FT trials was significantly increased compared to that for either the WR trials or the PV trials (one-tail t-test, max p = 8.8 × 10^−9^). Similar results were obtained for the other two WR- and PV-associated FAUPAs as shown in Fig. 8d. For each category of 4, 5 and 6 in Fig. 5, the corresponding trial-mean functional connectivity also demonstrated an expected behaviour for that category (Fig. 9). For example, for the FAUPAs associated with both WR and PV, the functional connectivity for either the WR trials or the PV trials was significantly increased compared to that for the FT trials (one-tail t-test, max p = 2.5 × 10^−5^) (Fig. 9e, left).

## Discussion

The activity of neural networks gives rise to simple motor behaviours as well as behaviours that are more complex. To understand the relationship between the network activity and human behaviours, it is necessary to identify functional networks specific for tasks and analyse the dynamic network activity when performing these tasks. To identify a task-specific network requires the identification of its constitutional elements. As the temporal variation of the signal time course of a task-associated FAUPA is the same across the FAUPA (Fig. 1b), the task-evoked underlying neural activity should be spatially coherent and temporally synchronized within that FAUPA, suggesting the role of a functional unit of the FAUPA for a particular neural computation of the task. Identifying all task-associated FAUPAs constitutes a network specific for that task (Fig. 3), and investigating the dynamic activity of these task-specific networks may enable us to understand the one-to-one relationship between the network activity and human behaviours via systematically manipulating task performance and measuring corresponding network activity change (Figs. 6–9).

A task-associated FAUPA may play a role of functional unit for the task-specific functional state and is essential for investigating the relationship of the task-specific network activity with the human behaviour. As different behaviours correspond to different brain functional states, the associated FAUPAs responsible for these different functional states should also vary accordingly. Thus, task-associated FAUPAs are functionally state-dependent. For example, when tapping both thumb and index fingers simultaneously we should expect to identify a task-associated FAUPA in the representation of these two fingers at the primary motor cortex, and, according to the somatotopic map^11^, this FAUPA should be smaller than the FT-associated FAUPA of tapping five fingers. These two task-associated FAUPAs reflect two different brain functional states corresponding to the two different behaviours. Some task-associated FAUPAs may be independent of functional states. For example, there may exist task-associated FAUPAs that only relate to the movement of fingers, regardless of tapping which finger (i.e., a non-somatotopic relation). Accordingly, identifying and analysing these task-associated FAUPAs and their task-specific network activity across the whole brain at a large-scale systems level are essential for investigating the relationship of the task-specific network activity with the human behaviour.

The determination of a FAUPA is based on the temporal similarity of the BOLD signal time courses within an area, and this determination is objective and automatic with no requirement of a priori knowledge of activity-induced BOLD responses^8^. The novel technique presented in this study for task-fMRI analysis is fundamentally different from the GLM^4,5^. To identify task-induced cortical activated areas, the GLM approach builds an ideal response and fits it with the signal time courses of voxels to determine the activated areas with a selected significance level. Consider the two FT-associated FAUPAs in the primary motor and somatosensory cortices in Fig. 1a. As their time courses are almost identical to each other (R = 0.973, p = 3.1 × 10^−61^), the GLM would identify these two FAUPAs as one activated cluster that combines them together (Fig. 2, left column). Although these two FAUPAs are adjacent to each other, they reflect two distinguishing sensory and motor functions, and therefore being able to identify them may be of critical importance for investigating these functions, showing a potential of the presented technique to improve the investigation of human brain functional organization.

It is well documented that the left primary motor cortex of the cerebrum controls the movement of the fingers of the right-hand and the right primary motor cortex controls the fingers of the left-hand, i.e., the somatomotor representations^11^. The cerebellum also plays an important part in movement control. It is well documented the decussate cerebrocerebellar circuit, i.e., the left cerebral cortex is connected to the right cerebellar cortex and the right cerebral cortex is connected to the left cerebellar cortex, respectively. The cerebrocerebellar circuit is a central nervous system circuit that mediates a two-way connection between the cerebral cortex and the cerebellum, and plays a crucial role in somatic functions concerning motor planning, motor coordination, motor learning, and memory^12,13^. Accordingly, tapping the fingers of the right-hand should activate the contralateral cerebrocerebellar circuit with respect to the cerebrum, as evidenced in both resting-state and task-fMRI studies^6,14–17^. However, to the best of our knowledge, for the first time the presented study provided indisputable evidence of the activation of the ipsilateral cerebrocerebellar circuit during the performance of each FT task (Fig. 3f,g, Table 1), though its functional role remains to be explored. Further studies are needed to replicate this finding and explore the functional role of this ipsilateral cerebrocerebellar circuit.

Many factors such as mood, level of alertness, motivation, neurological disorders, etc., can influence neural activity and task performance. A remarkable inter-subject variation in the task-induced activation from trial to trial was observed, reflected in the substantial variation in the total number of the task-associated FAUPAs from subject to subject for each FAUPA category (Table 2). When identifying the task-specific networks for each individual subject, the subject-dependent effects from these factors may be handled effectively by using the subject’s own signal time courses of the representative FAUPAs instead of using their corresponding ideal responses (Fig. 2, left and 2^nd^ columns). Its advantage was also reflected in the remarkable similarity in the signal time courses of the FT-induced activation across the whole brain (Fig. 3), yielding a more accurate activation map (Fig. 2, 2^nd^ column). This approach may be more suitable for clinical utility of fMRI such as using fMRI to aid pre-surgical planning^18^. As an example, we can use an identified FAUPA in the proximity of a brain tumour to determine its functional connectivity map across the whole brain, and this may aid to identify which function the area is associated. Doing so for each FAUPA surrounding the tumour may identify which FAUPA is connected with which functional network. Besides the inter-subject variation, a noticeable intra-subject variation in the activation from trial to trial was also observed. For example, when performing the FT task, the FT-induced activations in the left primary motor and somatosensory cortices varied from trial to trial (Fig. 1c,d). These two activations, however, were significantly correlated with each other for the eight FT trials (R = 0.9259, p < 0.001) (Fig. 1f, middle), suggesting a one-to-one relation between the activation and the task performance for each FT trial. This one-to-one relation may enable us to predict and verify the causal relationship between task performance and FAUPA activation via systematically manipulating task performance and assessing the corresponding FAUPA activation change. For example, moving the onset time or changing the duration of a FT task should produce a corresponding change to the onset time or shape of the task-induced FAUPA activation, respectively, and these predictions can be experimentally verified. The capability to characterize inter-subject variation with the signal time courses of task-associated FAUPAs may help to examine the variations across individuals and relate them to diseases. For example, a task-induced BOLD response in a cortical area may behave similarly for healthy people, but differently for patients who have difficulty of performing the task properly. Examining the similarity of the BOLD response in the same area between the healthy people and the patients may reveal the relationship of the changed BOLD response with the disease, but such a study is beyond the scope of this work.

The dynamic activity of a task-specific network may be characterized by (1) the dynamic activation of all FAUPAs within the network from trial to trial, and (2) the dynamic functional connectivity among these FAUPAs from trial to trial. The first one characterizes the dynamic task-evoked neural activity across the network, and the second one characterizes the dynamic co-activity across the network. The presented study provides evidence to demonstrate the dynamic activity of the three task-specific networks with the signal time courses of all the task-associated FAUPAs that constitute these networks (Fig. 5). First, the dynamic activation from trial to trial for each network is illustrated in Figs. 6 and 7. For example, for the FT network, when performing each FT task (trial 3, 6, 9, …), the activation of all FT-associated FAUPAs (i.e., category 3 in Fig. 5) in the FT network was substantially enhanced as evidenced in the group-mean activation in Fig. 6c, compared to that when performing each WR or PV task (trial 1, 2, 4, 5, …). All FAUPAs in categories 5, 6 and 7 in Fig. 5 were also activated when performing each FT task (Fig. 7b–d), but FAUPAs in categories 1, 2 and 4 in Fig. 5 were not activated (Figs. 6a,b and 7a). The dynamic activation of the other two networks showed a similar behaviour to that of the FT network. These results illustrate the dynamic activation patterns on the three networks from trial to trial that are associated with each task performance. Second, the dynamic functional connectivity from trial to trial is illustrated in Figs. 8 and 9. For the FT network, when performing each FT task, the functional connectivity among all FAUPAs in category 3 in Fig. 5 was substantially increased as evidenced in Fig. 8c. The functional connectivity among all FAUPAs in category 6 in Fig. 5 showed an expected behaviour, i.e., the functional connectivity was increased when performing either the FT or the PV task compared to that when performing the WR task (Fig. 9c). For all FAUPAs in category 5, the functional connectivity showed a similar behaviour to that for category 6 (Fig. 9b). The dynamic functional connectivity of the other two networks showed a similar behaviour to that of the FT network. These results illustrate the dynamic functional connectivity of the three networks from trial to trial that are also associated with each task performance.

A task network is composed of all FAUPAs that are associated with the task, i.e., every FAUPA is activated when the task is performed. Ideally, any identified task network should include all FAUPAs that do belong to the network but exclude all those that do not belong to it. In this pilot study as a proof of concept, an arbitrary threshold R = 0.8 (p = 5.5 × 10^−42^) was chosen to illustrate the capability of the presented technique in identifying task-specific networks (Table 3). To identify the FAUPAs for each FAUPA category, we selected the FAUPA with the signal time course that mostly resembled the ideal response of the category, and subsequently using the signal time course of this selected FAUPA as the reference function to identify the FAUPAs with the threshold R of 0.8. Note that, it may result in misidentified FAUPAs for a category if we simply use the ideal response of the category as the reference function to identify the FAUPAs because of the large R among some of the ideal responses (Table 3). For the same reason, it may also result in misidentified FAUPAs if we use the ideal response to select the FAUPA with the maximal R, and then use its signal time course to identify the FAUPAs without a validation. As the presented FAUPA approach identifies task-associated FAUPAs for each individual, it provides a means of testing the accuracy of the identification with properly varying the task and validating corresponding changes as predicted for precisely identifying the task-specific network without relying on arbitrarily choosing a threshold R or a significance level for a statistical model. Such identified task-specific networks may enable us more effectively to investigate the network activity with the human behaviour.

**Table 3:** Comparison of Pearson correlation coefficients among the seven task-induced ideal response time courses vs. that among the group-mean signal time courses of the seven selected task-associated FAUPAs (dashed-lines in Fig. 5).

R between the seven task-induced ideal response time courses							
Task	WR	PV	FT	WR_PV	WR_FT	PV_FT	WR_PV_FT
WR	1	−0.091	−0.091	0.674	0.674	−0.135	0.522
PV	−0.091	1	−0.091	0.674	−0.135	0.674	0.522
FT	−0.091	−0.091	1	−0.135	0.674	0.674	0.522
WR_PV	0.674	0.674	−0.135	1	0.400	0.400	0.774
WR_FT	0.674	−0.135	0.674	0.400	1	0.400	0.774
PV_FT	−0.135	0.674	0.674	0.400	0.400	1	0.774
WR_PV_FT	0.522	0.522	0.522	0.774	0.774	0.774	1
**R between the seven group-mean signal time series**							
Task	WR	PV	FT	WR_PV	WR_FT	PV_FT	WR_PV_FT
WR	1	0.232	−0.074	0.808	0.547	0.183	0.743
PV	0.232	1	−0.031	0.650	0.152	0.553	0.591
FT	−0.074	−0.031	1	−0.091	0.716	0.753	0.290
WR_PV	0.808	0.650	−0.091	1	0.443	0.389	0.860
WR_FT	0.547	0.152	0.716	0.443	1	0.721	0.730
PV_FT	0.183	0.553	0.753	0.389	0.721	1	0.675
WR_PV_FT	0.743	0.591	0.290	0.860	0.730	0.675	1

In summary, the novel technique presented here is capable of identifying task-specific networks that demonstrate precise locations of task-associated FAUPAs across the whole brain. The computed activity of these task-specific networks from trial to trial produces a dynamic pattern of the large-scale neural activity across the whole brain when performing these tasks. It relates the network activity with the human task performance, offering a means of testing the causal relationship between network activity and human task performance via systematically manipulating task performance and measuring corresponding network activity change.

## Methods

This is a follow-up study of our previous two studies^8,9^. This study analyzed the same set of the task-fMRI data. Accordingly, it used the same subjects, same task paradigm, same image acquisition, similar image preprocessing procedures, same algorithms for FAUPA determination, and same approaches for identifying task-associated FAUPAs. For more information please refer to our previous studies^8,9^.

### Subjects

Nine healthy subjects (5 male and 4 female, ages 21–55 years old) participated in the study. The Institutional Review Board at Michigan State University approved the study, and written informed consent was obtained from all subjects prior to the study. All methods were performed in accordance with the institution’s relevant guidelines and regulations.

### Task paradigm

The task paradigm consisted of a total of 24 task trials with 3 different tasks of word-reading (WR), pattern-viewing (PV) and finger-tapping (FT). Each trial comprised a 6-s task period followed by a 24-s rest period. During a WR task period, the subjects silently read the presented English word once. During a PV task period, they passively viewed the presented striped pattern. During a FT task period, they were visually cued to tap their right-hand five fingers as quick as possible in a random order. The presentation of the three tasks is interleaved. During the 24-s rest period, subjects were instructed to focus their eyes on a fixation mark at the screen center and try not to think of anything.

### Image acquisition

Functional brain images were acquired on a GE 3.0 T clinical scanner with an 8-channel head coil using a gradient echo Echo-Planar-Imaging pulse sequence (TE/TR = 28/2500 ms, flip angle 80°, FOV 224 mm, matrix 64 × 64, slice thickness 3.5 mm, and spacing 0.0 mm). Thirty eight axial slices to cover the whole brain were scanned, and the first three volume images were discarded. The visual stimuli were projected onto a vertical screen placed inside the magnet bore using a MR-compatible projection system, the stimulation presentation was controlled by a PC equipped with E-Prime, and a Button Response System with a pair of 5-button MR-compatible keypads was used to record subjects’ finger-tappings (Psychology Software Tools, Inc., Pittsburgh, PA). The participants viewed the screen through a mirror mounted on top of the head coil. For the participants who needed vision correction, MR-compatible lenses were used. Head movement was minimized by restraint using tape and cushions. Each participant first had a 12-min resting-state (rs) fMRI scan and then a 12-min task fMRI scan. Each scan yielded a total of 288 volume images (total time points N = 288). After the task-fMRI scan, T1-weighted whole-brain MR images were also acquired using a 3D IR-SPGR pulse sequence.

### Image preprocessing

Image preprocessing of the functional images was performed using AFNI (http://afni.nimh.nih.gov/afni)^19^, including (1) removing spikes; (2) slice-timing correction; (3) motion correction; (4) spatial filtering with a Gaussian kernel with a full-width-half-maximum of 4.0 mm; (5) computing the mean volume image; (6) bandpassing the signal intensity time courses to the range of 0.009 Hz – 0.08 Hz; and (7) computing the relative signal change (%) of the bandpassed signal intensity time courses. After these preprocessing steps, further image analysis was carried out using in-house developed Matlab-based software algorithms.

### FAUPA determination

A statistical model and Matlab-based software algorithms were developed and tested to determine FAUPA, and FAUPAs were identified and reported for both the rs-fMRI and task-fMRI^8,10^. The FAUPA determination involved the iterative aggregation of voxels dependent upon their intercorrelation, and the algorithms were described in detail in our previous study^8^. The determination consists of two major procedures. (1) Using a first statistical criterion the algorithm first identifies a stable region-of-interest (ROI) in which the signal time courses of all voxels show a similar temporal behaviour; and (2) Using a second statistical criterion it determines whether this stable ROI satisfies the condition of being a FAUPA by comparing the temporal behaviour of signal time course of the voxels within the FAUPA with those bordering the FAUPA.

### Comparison of the finger-tapping activation maps

We compared the FT-activated areas generated with the GLM and this newly introduced FAUPA method. To identify the FT-activated areas with the GLM, we first generated a FT-induced ideal response by convolving the temporal paradigm of the FT task alone with a hemodynamic response function, using the 3dDeconvolve program in AFNI with the convolution kernel SPMG3. We then computed its relative signal change time course by subtracting the signal time course with its mean and dividing that change with the mean. This ideal response time course was then used as the reference function to compute a voxel-by-voxel Pearson correlation coefficient (R) map for each participant in the original space. To identify the FT-activated areas with the FAUPA method, we first identified a FT-associated FAUPA in the finger-representative area in the left primary motor cortex (M1) for each participant, and then used the BOLD signal time course of the selected FAUPA as the reference to compute an R map in the original space. For group comparison, we converted all R maps to a standard template space (icbm452, an averaged volume of 452 normal brains) using AFNI. For each method, the mean R map was thresholded with R > 0.45 (N = 288, P < 2.2 × 10^−14^) to yield a FT-evoked activation map, and then we compared this activation map between the two methods.

### Identification of task-associated FAUPAs

We define a task-associated FAUPA as a FAUPA that is activated when performing the task. To identify those FAUPAs that were associated with a task, we first generated a task-induced ideal response by convolving the temporal paradigm of the task with the hemodynamic response function, and then computed its relative signal change time course. This ideal response time course was then used to identify those FAUPAs that were possibly associated with the task. There were three different tasks in this study, and therefore there were total of seven functional groupings (i.e., seven categories) among these tasks: (1) FAUPAs associated with the WR task alone; (2) those associated with the PV task alone; (3) those with the FT task alone; (4) those with both WR and PV tasks; (5) those with both WR and FT tasks; (6) those with both PV and FT tasks; and (7) those with all the three tasks. Accordingly, seven ideal response time courses were generated for these seven categories of task-associated FAUPAs. (Generally speaking, the task-induced responses may have different magnitudes for the three tasks, but in this study, for simplicity, we assume the magnitude of the ideal response for categories 4 to 7 is the same for all three tasks.) There are no overlaps in the task-evoked responses among the first three categories, but substantial overlaps for other categories. Table 3 tabulated the R-value among these seven ideal response time courses, and the maximum R is 0.774, showing that a chosen threshold R < 0.774 would likely misidentify a FAUPA between the two categories such as category 4 vs. 7. Accordingly, we chose R = 0.8 as the threshold for determining task-associated FAUPAs in this study. As task-induced responses may vary from subject to subject, for each subject, to identify a representative task-associated FAUPA for each category, we first computed the correlation of the ideal response time course with the signal time course of each FAUPA, and then sorted them with R. Then, we visually examined the BOLD signal time courses for those FAUPAs with large R values, and selected the FAUPA with a signal time course that resembled the ideal response the most as the representative task-associated FAUPA for the category and the subject. These signal time courses are almost perfectly correlated with their corresponding ideal responses (the minimum R = 0.87, the maximum p = 2.3 × 10^−49^), verifying the selection of these representative task-associated FAUPAs. To identify task-associated FAUPAs for each category and each subject, we used the signal time course of the representative FAUPA to compute its correlation with that of all other FAUPAs, and identified those FAUPAs with R > 0.8 as the task-associated FAUPAs for the category and the subject. For comparison, Table 3 also tabulated the R-value among the group-mean signal time courses of these seven selected FAUPAs, and most of them are comparable to those between the ideal responses, further validating these selected representative task-associated FAUPAs.

### Dynamic activity of task-specific networks

The dynamic network activity of a task-specific network may be characterized by the temporal changes of activation and functional connectivity of all FAUPAs within the network, i.e., the activation change of each FAUPA and the functional connectivity change of any paired FAUPAs within the network from trial to trial. A recent fMRI study of an achiasmic human visual cortex quantifies the relationship between the fMRI BOLD signal and neural response; the magnitude of a stimulus-induced BOLD response is proportional to approximately 0.5 power of the stimulus-evoked underlying neural response^20^. To quantify the activation change of a FAUPA from trial to trial, the mean of the squared relative signal changes over each trial period is computed to quantify the FAUPA activation during the trial period. Accordingly, for a given network, the activation changes of all FAUPAs within the network from trial to trial characterize the dynamic network activation. To quantify the functional connectivity change of any paired FAUPAs from trial to trial, the correlation of their signal time courses over each trial period was computed to quantify the functional connectivity during the trial period. Accordingly, for a given network, the functional connectivity changes of all paired FAUPAs within the network from trial to trial characterized the dynamic network functional connectivity. The collating changes of activation and functional connectivity as a function of task trial quantified the dynamic network activity from trial to trial.

## Data Availability

Both the original and processed fMRI images plus final research data related to this publication will be available to share upon request with a legitimate reason such as to validate the reported findings or to conduct a new analysis.

## References

[1] Ogawa S, Lee TM, Kay AR, Tank DW (1990). Brain magnetic resonance imaging with contrast dependent on blood oxygenation. Proc. Natl. Acad. Sci. USA..

[2] Kwong KK (1992). Dynamic magnetic resonance imaging of human brain activity during primary sensory stimulation. Proc. Natl. Acad. Sci. USA..

[3] Logothetis NK (2008). What we can do and what we cannot do with fMRI. Nature.

[4] Friston K (1995). Statistical parametric maps in functional imaging: A general linear approach. Hum. Brain Mapp..

[5] Worsley, K. J. In *FUNCTIONAL MRI: an introduction to methods*. (eds. Jezzard, P., Matthews, P. M. & Smith, S. M.) Ch. 14, 251–270 (Oxford University Press, 2001).

[6] Moritz CH, Haughton VM, Cordes D, Quigley M, Meyerand ME (2000). Whole-brain functional MR imaging activation from a finger-tapping task examined with independent component analysis. AJNR. Am J .Neuroradiol.

[7] Chen Z, Calhoun V (2018). Effect of Spatial Smoothing on Task fMRI ICA and Functional Connectivity. Front Neurosci.

[8] Huang J (2018). Human brain functional areas of unitary pooled activity discovered with fMRI. Sci. Rep..

[9] Huang J (2019). Greater brain activity during the resting state and the control of activation during the performance of tasks. Sci. Rep..

[10] Huang, J. Method and system for determining brain-state dependent functional areas of unitary pooled activity and associated dynamic networks with functional magnetic resonance imaging. *United States Patent and Trademark Office, PCT Application (PCT/US2018/019819), filing data: February**27* (2018).

[11] Penfield W, Boldrey E (1937). Somatic motor and sensory representation in the cerebral cortex of man as studied by electrical stimulation. Brain.

[12] Allen GI, Tsukahara N (1974). Cerebrocerebellar communication systems. Physiological Reviews.

[13] Benagiano V (2018). The functional anatomy of the cerebrocerebellar circuit: A review and new concepts. J. Comp. Neurol..

[14] Grodd W, Hulsmann E, Lotze M, Wildgruber D, Erb M (2001). Sensorimotor mapping of the human cerebellum: fMRI evidence of somatotopic organization. Human Brain Mapping.

[15] Buckner RL, Krienen FM, Castellanos A, Diaz JC, Yeo BT (2011). The organization of the human cerebellum estimated by intrinsic functional connectivity. J. Neurophysiol.

[16] Wiestler T, McGonigle DJ, Diedrichsen J (2011). Integration of sensory and motor representations of single fingers in the human cerebellum. Journal of Neurophysiology.

[17] Stoodley CJ, Valera EM, Schmahmann JD (2012). Functional topography of the cerebellum for motor and cognitive tasks: An fMRI study. Neuroimage.

[18] Hou BL, Bhatia S, Carpenter JS (2016). Quantitative comparisons on hand motor functional areas determined by resting state and task BOLD fMRI and anatomical MRI for pre-surgical planning of patients with brain tumors. Neuroimage Clin.

[19] Cox RW (1996). AFNI: software for analysis and visualization of functional magnetic resonance neuroimages. Comput Biomed Res.

[20] Bao P, Purington CJ, Tjan BS (2016). Using an achiasmic human visual system to quantify the relationship between the fMRI BOLD signal and neural response. eLIFE.

